# Performance of non-invasive prenatal testing for trisomies 21 and 18 in twin pregnancies

**DOI:** 10.1186/s13039-018-0392-2

**Published:** 2018-08-22

**Authors:** Jiexia Yang, Yiming Qi, Yaping Hou, Fangfang Guo, Haishan Peng, Dongmei Wang, O. Y. Haoxin, Yixia Wang, Huajie Huang, Aihua Yin

**Affiliations:** 1 Prenatal Diagnosis Centre, Guangdong Women and Children Hospital, Guangzhou, 511400 Guangdong China; 2 Maternal and Children Metabolic-Genetic Key Laboratory, Guangdong Women and Children Hospital, Guangzhou, 511400 Guangdong China

**Keywords:** Non-invasive prenatal testing (NIPT), Twin pregnancy, Trisomy 21, Assisted reproductive techniques (ART), Amniocentesis

## Abstract

**Background:**

Cell-free fetal DNA in maternal plasma represents a source of fetal genetic material that can be sampled noninvasively. There are ample studies confirming the accuracy of NIPT in singleton pregnancies, but there is still relatively little studies demonstrate the feasibility and clinical application of a NIPT for fetal aneuploidy screening in twin pregnancies.

**Results:**

In this study, we have finished 432 twin pregnancies screening by NIPT. There were 4 double chorionic dichorionic diamniotic (DCDA) cases of true positive NIPT results, including 1of T18 and 3 of T21, and 1 monochorionic diamniotic (MCDA) cases of true positive NIPT results, including 1of T21. The combined false-positive frequency for trisomies 21, 18 was 0%. Furthermore, there were 2 cases of false positive NIPT results, including 1 of T7 and 1 of sex chromosome aneuploidy. There was no false negative case, which gave a combined sensitivity and specificity of 100 and 99.53% respectively.

**Conclusion:**

Our study demonstrated NIPT performed well in the detection of trisomy 21 in twin pregnancy. It is feasible and clinical applicable of NIPT for fetal aneuploidy screening in twin pregnancies. But, it needs a large number of clinical samples to demonstrate the applicability of other chromosomal abnormalities besides trisomies 21 and 18 in both singleton pregnancies and twin pregnancies.

## Background

Non-invasive prenatal testing for aneuploidy by cell-free DNA (cfDNA) has been available clinically for over several years. In singleton pregnancies, cfDNA analysis of maternal blood provides effective screening for trisomies 21, 18 and 13 with respective detection rates of about 99, 97 and 92%, at a combined false-positive rate (FPR) of 0.4 [1]. Such high performance of screening has been reported for both high-risk pregnancies and in the general population [2–4].

However, multiple births now account for 3% of all births worldwide [5, 6]. The rate of twin birth increased by more than 75% in the United States from 1980 to 2009 [7]. Similar trends have been observed in Western Europe and other countries [5]. Traditionally, both the risk for aneuploidies and the risk of miscarriage from invasive testing are higher in twin pregnancies than in singletons [8, 9]. With a high proportion of twin births thought to originate in women undergoing assisted reproductive technology (ART), the use of non-invasive prenatal testing (NIPT) to screen for fetal aneuploidy is especially desirable. Preliminary data have suggested that NIPT is a feasible test option for twin gestations [10, 11]. Currently, due to the lack of data in twins, professional societies have called for more researches on NIPT performance in twin gestations [12–14].

However, in twin pregnancies cfDNA testing is more complex than in singleton pregnancies because if a trisomy occurs in a dizygous twin pregnancy, usually only one of the fetuses is affected, and the contribution of cfDNA of the two fetuses into the maternal circulation can vary by nearly two-fold [15, 16]. If the fetal fraction of the affected fetus is below the threshold of 4% necessary for successful cfDNA analysis, but there is a high contribution from the normal cotwin. Consequently, the complexity of the fetal fraction in twin gestations has raised concerns about a potentially increased false-negative rate of NIPT in twin gestations. It is then crucial that each twin contributes enough cell-free fetal DNA (cffDNA) to discriminate between aneuploid and euploid pregnancies.

Professional societies and others have called for more studies on NIPT performance in twin gestations [13, 14], and an increasing number of clinical data are showing that the sensitivity and specificity for the detection of fetal trisomy 21 by NIPT in twin pregnancies—monozygous as well as dizygous—are comparable to those in singleton pregnancies [16, 17].

Cell-free DNA testing in twin pregnancies is more complex, because the 2 fetuses could be either monozygotic or dizygotic, in which case only 1 fetus is likely to have aneuploidy when present. In this study, we have finished 432 twin pregnancies screening by NIPT. There were 5 cases of true positive NIPT results, in which only 1 fetus was aneuploidy of the 5 cases. At last, we discussed the feasibility and clinical application of NIPT for fetal aneuploidy screening in twin pregnancies.

## Methods

### Samples collection

In this study, 432 twin pregnancies underwent screening for trisomies 21, 18 and 13 by cfDNA testing between January 2015 and December 2016. All patients received detailed pretest counseling and provided written informed consent for the test.

### Maternal plasma DNA sequencing

Blood samples from women pregnant with twin gestations have been collected for NIPT. From each pregnant woman, 5 mL of peripheral blood was obtained in an ethylene diamine tetraacetic acid-anticoagulated tube before invasive procedures, and plasma was separated within 8 h following a double-centrifugation protocol. All subsequent procedures, including cell-free DNA isolation, library construction, and sequencing, were performed according to instructions of JingXin Fetal Chromosome Aneuploidy (T21, T18, T13) Testing Kits (CFDA registration permit No. 0153400300).

### Bioinformatics analysis for the detection of trisomies 21 and 18

Based on our previous study, we developed a technique that uses the read length to estimate the concentration of fetal cfDNA in maternal plasma by sequencing [18]. The fetal DNA concentration was calculated as a quality control, as described in Yin’s paper [18]. Combined GC-correction and Z-score testing methods were used to identify fetal autosomal aneuploidy for trisomy as described in Liao’s paper [19]. Z score range from − 3 to 3 was considered to indicate a low risk for a trisomy chromosome [20], and if Z score were > 3, the sample was in the high-risk zone.

### Karyotyping analysis

Invasive sampling was performed for high risk cases. The metaphase chromosome Gbanding karyotyping was performed at a level of 320 to 400 bands. The results of karyotyping were used as the gold standard to calculate the sensitivity and specificity of sequencing-based NIPT. In this study, 95% confidence intervals were evaluated on the basis of standard normal distribution.

## Results

### Study population

In this study, there were 432 twin pregnancies underwent screening for NIPT. Basic characteristics of the study population are shown in Table 1. 58.5 and 77.9% of pregnant women were younger than 35 years old. There were 337 pregnancies of double chorionic dichorionic diamniotic (DCDA), and there were 247 pregnancies ofartificial reproductive technology (ART) of those 337 cases. There were 95 pregnancies monochorionic diamniotic (MCDA), and there were 92 pregnancies of ART of those 95 cases.

**Table 1:** Demographic characteristic of pregnant women undergoing NIPT

Characteristic	DCDA(*n* = 337)	MCDA(*n* = 95)
Maternal age (years)		
< 35(n, %)	197(58.5)	74(77.9)
> =35(n, %)	140(41.5)	21(22.1)
Gestational age NIPT		
9-13 week(n, %)	47(13.9)	14(14.7)
14-27 week(n, %)	278(82.5)	72(75.9)
> =28 week(n, %)	12(3.6)	9(9.5)
Type of pregnancy		
Artificial reproductive technology	247(73.3)	92(96.8)
Natural pregnancy	90(26.7)	3(3.2)

*DCDA* Dichorionic diamniotic, *MCDA* Monochorionic diamniotic

### NIPT positive of fetal trisomies 21 and 18

There were 5 of true positive cases in the 432 twin pregnancies, including 1 case of trisomies 18 and 4 cases of trisomies 21. Clinical details of the 5 cases were summarized in the Table 2. Maternal age of case 1 was 31, and all of the T21 cases were advanced maternal age. The gestational age of the 3 cases was from 16 to 19 weeks, and all of the cases were pregnancy with artificial reproductive technology except case 5. There were 2 of false positive cases in the 432 twin pregnancies, including 1 case of T7 and 1 case of sex chromosomal aneuploidy. Table 3 summarized the clinical information of these 2 false positives cases. Both cases were pregnancies with artificial reproductive technology. Fetal fractions of the 5 true positives cases were 9.0, 7.5, 6.1, 30.6 and 21.2, and false positive cases were 7.6 and 19.7. The Z scores were > 3 of each case (Tables 2, 3). All of the 7 cases were identified as high risk sample, which were suggested to take invasive test for further diagnosis.

**Table 2:** Clinical details of the 5 cases with fetal trisomies 18 and 21

Placentation	Case	Maternal age	Gestational age	Conception	Fetal fraction	BMI	NIPT Result	Karyotyping
DCDA	Case 1	31	19	ART	9	25	T18	Normal/T18
	Case 2	37	18	ART	7.5	22	T21	Normal/T21
	Case 3	35	16	ART	21.2	23	T21	Normal/T21
	Case 4	36	16	ART	30.6	22	T21	Normal/T21
MCDA	Case 5	37	16	NP	6.1	27	T21	Normal/T21

*DCDA* Dichorionic diamniotic, *MCDA* Monochorionic diamniotic, *ART* Artificial reproductive technology, *NP* Natural pregnancy

**Table 3:** Clinical details of false positive NIPT results

Placentation	Case	Maternal age	Gestational age	Conception	Fetal fraction	BMI	NIPT Result	Karyotyping
DCDA	Case 6	26	14	ART	7.6	27	T7/ Normal	Normal
	Case 7	24	18	ART	19.7	20	47,XXX	NA

*DCDA* Dichorionic diamniotic, *MCDA* Monochorionic diamniotic, *ART* Artificial reproductive technology, *NP* Natural pregnancy. *NA* No abnormalities

### Karyotyping results

**Fig. 1:**
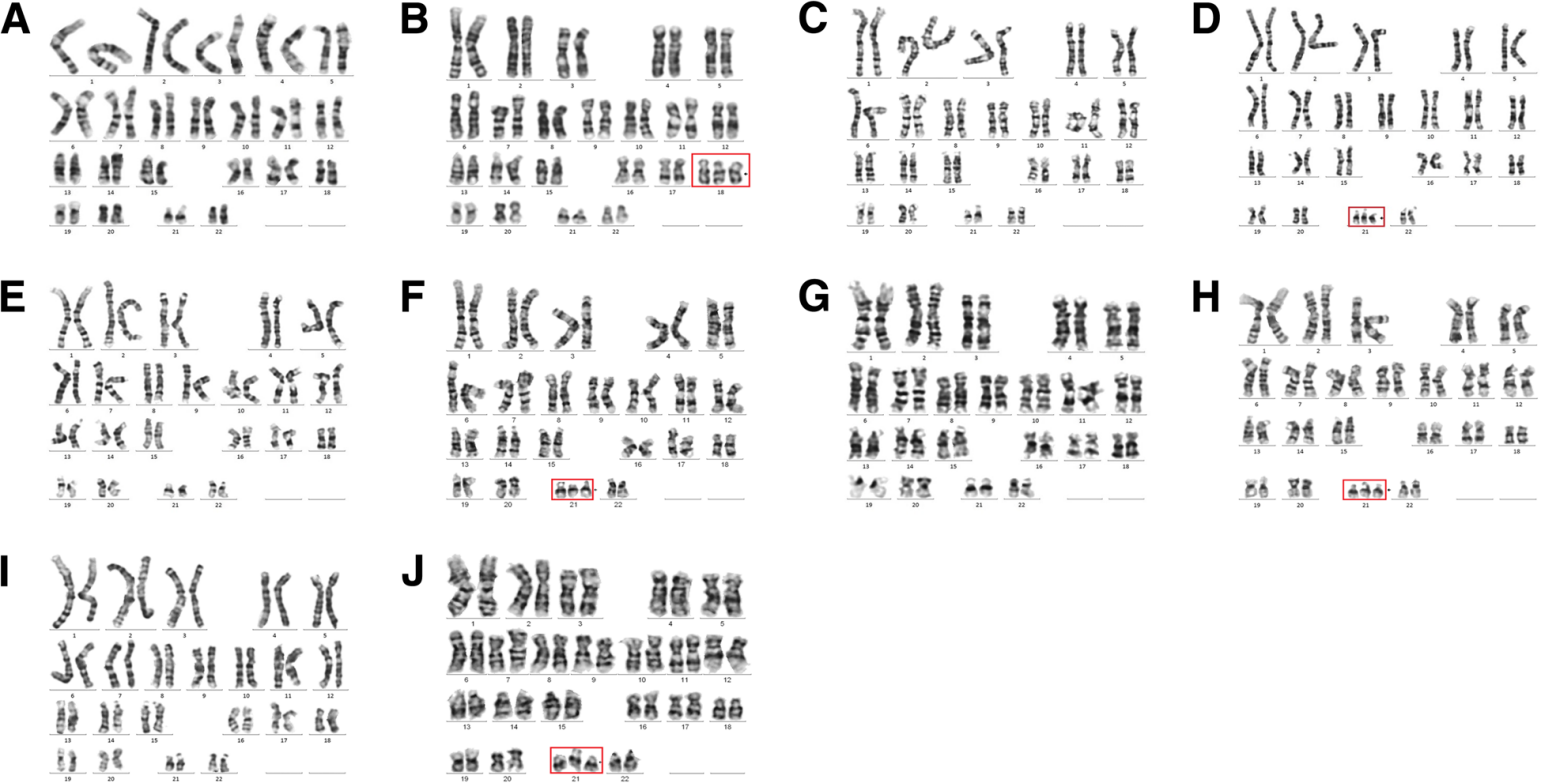
Karyotyping results of 3 cases. **a** and **b**, case 1, Normal/T18; **c** and **d**, case 2 Normal/T21; **e** and **f**, case 3, Normal/T21; **g** and **h**, case 4 Normal/T21; **i** and **j**, case 5 Normal/T21. Triploid chromosomes have been outlined with red line

Invasive sampling was performed for high risk cases, and karyotyping results were obtained in those 6 cases. Cases 7 refused to perform prenatal diagnosis. One of the fetuses of case 1 was trisomies 18, and the other fetus was normal. One of the fetuses of case 2, case 3, case 4 and case 5 was trisomies 21, and the other was normal. NIPT results showed the 5 cases were high risk, which was consistent with karyotyping results. In addition, karyotyping of case 6 was normal, which was discordance with NIPT result. Prenatal diagnosis proved case 6 was false positive of T7 (Fig. 1, Table 2 and Table 3).

### Follow-up investigations

Fetal reduction operation was performed in those true positive cases of trisomy 21 and trisomy 18. Pregnant woman of case 7 refused prenatal diagnosis, and decided to continue the pregnancy. At last, two normal fetuses were delivered. The remaining 425 cases were classified as negative. Pregnancy outcome was available in 393 cases, whereas the remaining 32 cases were lost to follow up. On the one hand, for DCDA cases, there were 301 pregnancies with normal fetus and a fetus of intrauterine death, and one of the twins induced labor because of an abnormal heart showed by ultrasonography. In addition, there are 3 cases of spontaneous abortion pregnancy, and 3 of termination of pregnancy. On the other hand, for MCDA cases, there were 82 pregnancies with normal fetus, and a fetus of intrauterine death. In addition, one fetus died after birth.

### NIPT performance

Accuracy is a very important parameter of NIPT, hence in this population of 432 twin pregnancies, karyotype or live birth feedbacks were obtained to compare the false negative and false positive results of the NIPT predicted results. Further calculation of NIPT sensitivity and specificity was based on this subgroup of the population with outcome data available. In total, we received 373 feedbacks. There were 5 true positive cases, 2 false positive cases and none false negative case, resulting the total sensitivity and specificity were 100 and 99.53% respectively.

## Discussion

Although NIPT performance has been extensively studied in singleton pregnancies, its efficacy is rarely reported in twin pregnancies. Here, we demonstrate the feasibility and clinical application of NIPT for fetal aneuploidy screening in twin pregnancies. In this study, we have finished 432 twin pregnancies screening by NIPT. There were 4 DCDA cases of true positive NIPT results, including 1of T18 and 3 of T21, and 1 MCDA cases of true positive NIPT results, including 1of T21, and there were 2 cases of false positive NIPT results, including 1 of T7 and 1 of sex chromosome aneuploidy. There was no false negative case, which gave a combined sensitivity and specificity of 100 and 99.53% respectively.

Conventional serum screening approaches have relatively high false-positive rates [21] in twin pregnancies and often cannot provide a result for trisomies 18 or 13 when compared with singletons. Therefore, there is a need for an accurate non-invasive method for fetal aneuploidy detection of trisomies 21, 18 and 13 in twin pregnancies. Cell-free fetal DNA in maternal plasma represents a source of fetal genetic material that can be sampled noninvasively. There are ample studies confirming the accuracy of NIPT in singleton pregnancies, but there is still relatively little studies demonstrate the feasibility and clinical application of a NIPT for fetal aneuploidy screening in twin pregnancies. Mar Gil et al. demonstrated cfDNA testing in twins was feasible but the reporting rate of results is lower than in singletons due to a lower fetal fraction [10]. Bevilacqua et al. demonstrated in twin pregnancies screening by cfDNA testing is feasible, but the failure rate is higher and detection rate may be lower than in singletons [11]. The last study of Fosler’s demonstrated NIPT performed well in the detection of trisomy 21 in twin pregnancy, NIPT for fetal Down syndrome in twin pregnancy is likely to be as accurate as in singleton pregnancies [22]. Prenatal screening is important to ART pregnancies, because couples opting for ART and the conceived fetuses have higher rate of chromosome abnormalities comparing to general population [23–25]. Furthermore, it is a particularly desirable option for ART patients for whom fear of procedure-related loss is heightened due to difficulties achieving pregnancy.

Twins can be either identical or fraternal. Therefore, it is of clinical importance to be able to distinguish the different types of twins for prenatal diagnosis. In our study, only one of the fetuses was affected and another was normal of all the true positive cases. Over half of the cases in DCDA group were ART pregnancies, and the proportion of ART pregnancies in MCDA group reached 96%. In DCDA group, our NIPT identified 4 trisomic twin pregnancies with 2 false positive and no negative. In group MCDA, evaluation of the 95 reported clinical twin samples revealed 1 with aneuploidy detected or suspected, no reported false positive in the aneuploidy detected sample and no reported false negative. Monochorionic twins are generally considered to be genetically identical, thus presenting a difficult counseling scenario when they have discordant anomalies [26]. Monozygotic twins with discordant prenatal presentations are a rare but known phenomenon with multiple potential explanations. The formation mechanism of MCDA that one fetuses has normal karyotype and another with trisomy may be as follows: (1) In meiosis, the chromosomes do not separate to form a gametes of 24 chromosomes, which combine with a normal gamete to produce a trisomic zygote, resulting in the formation of a trisomy rescue after the formation of a twin, and the random loss of an extra chromosome. A third of the fetus may be a single parent diploid fetus, the other child did not have trisomy to save itself, and continues to develop into trisomy; (2) The fertilized egg is a normal diploid. After the formation of the twin embryo, the chromosome is not separated during mitosis. Two cell lines, trisomy and monomeric are formed. The monomeric cell line dies because of its mortality, and the trisomy cell line develops into a trisomy; the other one continues to develop into a normal fetus.

Our study had finished 432 twin pregnancies screening by NIPT, there was no false negative case and the combined false-positive frequency for trisomies 21, 18 was 0%. But we had found 2 false positive cases, including 1 of T7 and 1 of sex chromosome aneuploidy. Case 6 had performed amniocentesis to diagnose, and amniocentesis confirmed karyotype of the twins was normal. Pregnant women of case 7 refused to take interventional prenatal diagnosis, she chose ultrasound examination regularly and ultrasound showed no abnormalities. At last, she delivered normal twin babies. False positives for NIPT can occur for a number of reasons, including maternal mosaicism [27, 28], maternal cancer [29], a demised twin [30, 31], or most commonly, confined placental mosaicism [32, 33]. NIPT reflects the genomic constitution of the placenta, not of the fetus itself and feto-placental discrepancy can cause false-positive (trisomy) NIPT results [34]. Chromosomal loss in trisomy (trisomy rescue) to generate a disomic fetus can cause confined placental mosaicism and/or feto/placental mosaicism. After trisomy rescue event, there is a risk of fetal uniparental disomy (UPD) [35], which is a strength of NIPT. In this study, we speculated that UPD may be a cause of false positive T7. UPD could lead to clinically significant conditions by producing either homozygosity for recessive mutations or aberrant patterns of imprinting, but UPD cannot be detected by conventional karyotyping, nor can it be detected by the current method of NIPT, so we recommend the baby to do more testing. Meanwhile, it needs a large number of clinical samples to demonstrate the applicability of NIPT in the detection of other chromosomal abnormalities besides trisomies 21, 18 and 13 in both singleton pregnancies and twin pregnancies.

## Conclusion

It may be argued that NIPT is not good enough because if it is positive, we still do not know which of the two fetuses is affected. But, for the far majority of cases in which both fetuses are normal, the NIPT will enable the couple to avoid any invasive test, because of the high sensitivity and specificity. NIPT enables the selection of almost only the affected pregnancies for invasive test. Recently, there were several publications suggested the possible for Non-invasive determination of zygosity [15, 36], and I believe it is possible to determine which of the two fetuses is affected in a screened positive case by NIPT in the near future.

## Abbreviations

ART: Assisted reproductive technology
cfDNA: Cell free DNA
cffDNA: Cell-free fetal DNA
DCDA: Double chorionic dichorionic diamniotic
GA: Gestational age
MCDA: Monochorionic diamniotic
NIPT: Non-invasive prenatal testing
NP: Natural pregnancy
T18: trisomy 18
T21: trisomy 21
